# An Australian View of Pensions

**Published:** 1905-07-01

**Authors:** 


### AN AUSTRALIAN VIEW OF PENSIONS.

An ex-secretary of the Children's Hospital, Melbourne, who broke down in the performance of his duties and became utterly incapacitated for work, has for some years been in receipt of a pension of £200 a year from the hospital. In his last report on the hospital, the inspector of charities (Mr. F. T. Short) advised the Cabinet that he thought this a misuse of the money granted to the hospital, and stated that he was strongly of opinion that the pension should be withdrawn. Failing that, he recommended that the Government subsidy (at present £500) should be withheld from the hospital. Yesterday a deputation from the hospital waited upon the Premier (Mr. Bent) to ask that this recommendation should not be followed.

Mr. Frank Madden (Speaker of the Legislative Assembly) said that the officer concerned was for over 20 years secretary of the hospital. His schemes for collecting money benefited the hospital immensely. He was now very ill. It would be unjust for the Government to withdraw its subsidy because the committee was doing its duty by this poor man.

Mr. R. Murray Smith and Mr. R. J. Larking (members of he finance committee of the hospital) eulogised the work done by the previous secretary. So much work was being done gratuitously that the hospital was now paying only £400 where it formerly paid £700. Mr. Murray Smith denied that the pension was a misuse of the revenue of the hospital. It was a laudable use of a small portion of it.

Mr. Bent.—Suppose I allowed it to all the hospitals?

Mr. Madden.—It was done in one case at the Melbourne Hospital. The pensioner is dead now.

Mr. Bent.—If you like, I will take it to the Cabinet. I won't do it myself. I don't believe in pensions. People are paid while they do the work.

Mr. Murray Smith.—Well, we shall regretfully have to do without the grant.

Mr. Madden.—He has a growing family.

Mr. Bent.—Let his family do as mine did—go to work. I will advise the Cabinet against it, but I am only one of ten.

Mr. Larking.—Would you advocate our turning him out into the street to apply for an old-age pension? The public would not approve of that.

Mr. Bent.—I think the public has had enough pensions. I know I have. What is the use of my preaching about £1,000 a day if I don't stop it wherever I can?

Mr. Murray Smith.—The ladies of the committee are under a legal liability to this man now.

Mr. Bent.—Well, I am knocking this precedent out. If the gentleman at the Melbourne Hospital had been alive I would have knocked that out too. I will knock a good many of them out.

The deputation then withdrew.

